# Determination of pharyngeal airway space volumes and their correlation with gender and age: A retrospective CBCT study

**DOI:** 10.6026/9732063002002004

**Published:** 2024-12-31

**Authors:** Naveen Kumar Shetty, Mandavi Waghmare, Sandeep Pagare, Abhishek Mukherjee, Reema Manoj

**Affiliations:** 1 Department of Oral Medicine & Radiology School of Dentistry, DY Patil (Deemed to be University) Navi Mumbai, Maharashtra, India

**Keywords:** Airway volumes, age, gender, pharyngeal airway space

## Abstract

An analysis of full skull cone beam computed tomography (CBCT) scans of 180 patients to measure the upper airway volumes and analyze correlations with age and gender is of interest. Results showed that 45% of patients had reduced airway volume, 21.7% had increased volume and 33.3% had normal volumes. Male patients had significantly higher volumes of the nasopharyngeal (NPV) and total upper airway volume (TV) values, with a notable age-related decrease in oropharyngeal (OPV). The study highlights gender and age as significant factors influencing airway volumes.

## Background:

The upper airway consists of the nasal and oral cavities, pharynx and larynx, connecting the oral and nasal cavities to the larynx and esophagus [1]. It includes three main components: the nasopharynx (mainly respiratory), oropharynx (containing five layers and the palatine tonsil) and hypopharynx [2]. The base of the tongue is crucial for oropharyngeal cancer symptoms and the vallecula helps prevent saliva and debris from entering the larynx, reducing foreign body aspiration risk [3]. The epiglottis prevents aspiration during swallowing, while the pharynx/laryngopharynx divides into the larynx and esophagus, including the posterior pharyngeal wall and pyriform sinuses [4]. The oral cavity, pharynx and larynx facilitate swallowing, with the laryngopharynx transporting air, water and food [5]. The soft palate separates the nasopharynx and oropharynx, and the mandible's position affects pharyngeal airway dimensions [6]. Upper airway assessment employs various imaging techniques such as acoustic reflection, fluoroscopy, nasopharyngeal endoscopy, cephalometry, computed tomography (CT), magnetic resonance imaging (MRI) and cone beam computed tomography (CBCT) [7]. Acoustic reflection measures the upper airway area and fluoroscopy evaluates the airway in different states [8]. Nasopharyngeal endoscopy assesses nasal passages and vocal cords. Cephalometry standardizes head and neck radiographs to examine bony and soft tissue structures [9, 10]. CT provides multi-slice images for volume reconstruction, while MRI offers high-resolution imaging without radiation, which benefits obstructive sleep apnea (OSA) patients [11]. CBCT, or digital volume tomography (DVT), uses divergent X-rays for imaging, offering advantages like lower costs and reduced radiation exposure. It allows precise imaging of the maxillofacial region and facilitates nerve and arch tracing [12]. Parameters assessed with CBCT include airway patency, symmetry, total upper airway volume (TV) and volumes of the nasopharyngeal (NPV), oropharyngeal (OPV) and hypopharyngeal (HPV) airways [13]. Recent studies have utilized segmentation software such as Ondem and 3D, Orca, Dolphin, Simple ITK, ITK-SNAP and MIMICS for airway evaluation. There is also literature on correlations between gender and age with upper airway dimensions on CBCT [14, 15, 16- 17, 21, 22, 23-24]. Therefore, it is of interest to assess pharyngeal airway space volumes using only CBCT and segmentation software to calculate total airway volume and examine changes (increase, decrease, or regular) concerning gender and age. We are specifically interested in measuring the volumes of the nasopharynx, oropharynx, hypopharynx and total airway volume and correlating these with gender and age.

## Methodology:

This retrospective study was approved by the Institutional Review and Ethics Board of DY Patil University School of Dentistry, Navi Mumbai (Approval No. IREB/2023/OMR/08 on July 31, 2023). Analyzed CBCT scans to assess pharyngeal airway volumes. Sample size estimation was based on a formula from the literature, resulting in 180 CBCT scans (90 males and 90 females) of patients aged 18-72 years. Scans were acquired using a Kodak Carestream CS9600 device with a field of view (FOV) of 16x10 cm.

## Inclusion and exclusion criteria:

Inclusion criteria involved patients aged 18-72 who were advised to undergo full-skull CBCT scans. Exclusion criteria included scans of patients under 18 years, those with recent maxillo-mandibular surgeries or airway interventions (*e.g.*, tracheostomy), those with a history of orthodontic/orthognathic treatment, conditions such as severe nasal septal deviation, diffuse sinusitis, or nasopharyngeal carcinoma and Skeletal Class II or III malocclusion. Scans with motion or beam-hardening artifacts were also excluded.

## Imaging and analysis:

Scans were saved in DICOM format and analyzed using CS 3D Imaging Software (v 8.0). ITK-SNAP software (v 4.2.0) was used for volumetric assessment to segment nasopharynx, oropharynx and hypopharynx volumes using predefined anatomical landmarks. Each volume was calculated in cubic millimetres and converted to cubic centimetres. The total pharyngeal airway volume range was defined as 20-23 cm^3^. Reductions in airway volume were classified as follows: mild (0-10%), moderate (11-20%), moderate-to-severe (20-30%) and severe (≥30%).

## Data collection and statistical analysis:

Data, including patient demographics and airway volumes, were recorded in MS Excel (v 2019) and included metrics such as Nasopharynx Volume (NPV), Oropharynx Volume (OPV), Hypopharynx Volume (HPV), Total Airway Volume (TV) and percentage reductions where applicable. Statistical analysis was performed using SPSS (v 26.0, IBM). Intergroup comparisons between two groups (male and female) were conducted with t-tests and Pearson correlation was used for bivariate analysis. Significance was set at p < 0.05 with a study power of 80% (α = 5%, β = 20%). Statistical significance was noted as follows: *p < 0.05 (significant), **p < 0.01 (essential) and #p > 0.05 (non-significant).

## Results:

**Figure 1:**
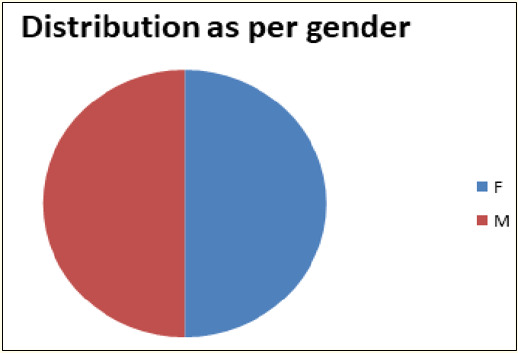
Distribution of patients in our study based on gender

**Figure 2:**
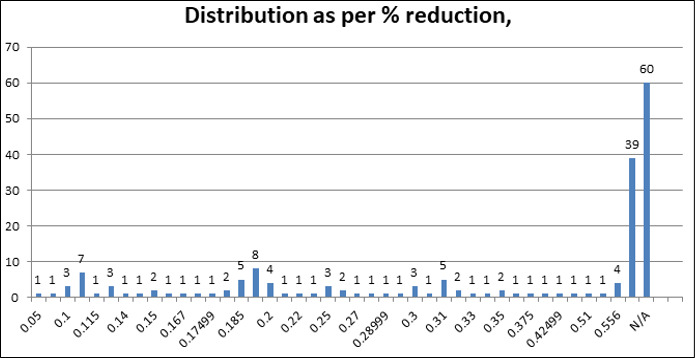
Distribution of patients based on reduction in airway volume

In Table 1 above, of a total sample size comprising 180 patients, there were patients in the age range of 20-72 years with a mean age of 42.61 years & a standard deviation (SD) of 13.66 was noted. The minimum NPV was 3 cm^3^, with the highest NPV value being 13.3 cm^3^& mean NPV being 7.29 cm^3^. The minimum OPV was 2 cm^3^, with the highest OPV value being 19 cm^3^ with the mean OPV value being 8.42 cm^3^. The minimum HPV was 1.4 cm^3^ & the highest HPV value was 11.4 cm^3^, with the mean HPV value being 5.26 cm^3^. The minimum TV was 9.4 cm^3^ with the maximum TV value being 40.1cm^3^& the mean TV value was noted to be 20.98cm^3^. Figure 1 shows the equal distribution of male & female patients in our study (90 each), with a total sample size of 180. Figure 2 shows the distribution of patients based on reduction in volume(R), as well as those with an increased airway volume (INC) and those with normal airway volumes (N/A). About 39 patients presented with an increased airway volume & 60 patients presented with a normal airway volume (N/A). The percentage of reduction in total airway volume ranged from as low as 5 % to as high as 55.6%, as highlighted above. Thirty-nine patients (21.7%) presented with an increase in airway volume, while 60 patients (33.3%) presented with normal airway volume.81 patients (45%) presented with a reduction in overall airway volume.

## Table 2 denotes:

There was a statistically highly significant difference seen for the values between the groups (p<0.01) for NPV (nasopharyngeal volume) with higher values in male patients. There was a statistically significant difference seen for the values between the groups (p<0.05) for TV (Total airway volume) with higher values in male patients. There was a statistically non-significant difference seen for the values between the groups (p>0.05) for OPV (oropharyngeal volume) & HPV (hypopharyngeal volume). The Table 3 shows an inter-group comparison of airway analysis based on gender. Twenty-five males & 14 females presented with increased airway volumes. Thirty-one females & 29 males presented with normal airway volumes. Thirty-six males & 45 females presented with reduced airway volumes, respectively.

**Figure 3:**
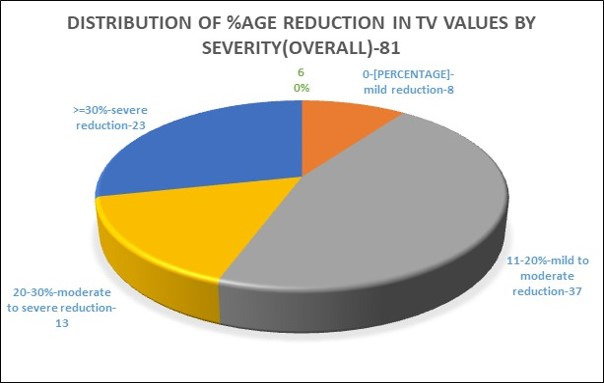
Distribution of total airway volume (TV) values based on percentage of volume reduction by severity (overall)

The pie chart (Figure 3) shows 81 patient scans that showed a reduction in total airway volume; we have proposed to classify these in the following order based on percentage reduction of volume & severity overall as mentioned below

8 patients showed 0-10% reduction (mild reduction)

37 patients showed 11-20% reduction (moderate reduction)

13 patients showed a 21-30% reduction (moderate to severe reduction)

23 patients showed >=30% reduction (severe reduction)

**Figure 4:**
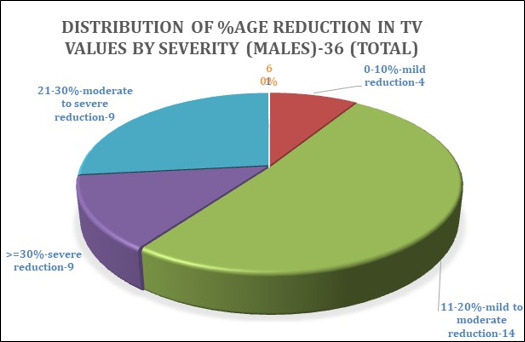
Distribution of Total Airway Volume (TV) values based on percentage in volume reduction by severity (males)

The pie chart (Figure 4) shows 36 male patient scans that showed reduced total airway volumes. Based on the percentage of reduction, we proposed to classify these in the following order, as mentioned earlier

4 patients showed a 0-10% reduction in TV values (mild reduction)

14 patients showed 11-20% reduction (moderate reduction)

9 patients showed a 21-30% reduction (moderate to severe reduction)

9 patients showed >=30% reduction (severe reduction)

**Figure 5:**
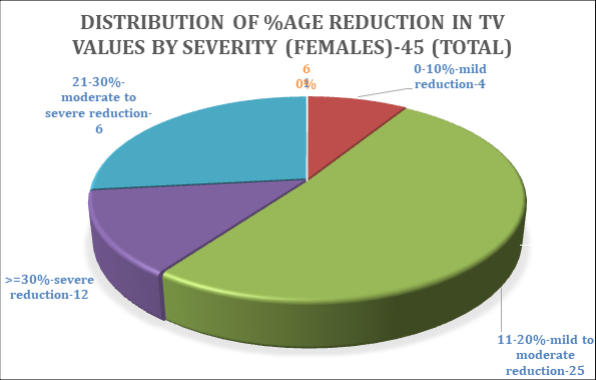
Distribution of TV values based on percentage in volume reduction by severity (females)

The pie chart (Figure 5) shows 45 female patient scans showing reduced airway volumes. As mentioned earlier, we proposed classifying these scans in the following order based on the percentage reduction in volume

4 patients showed 0-10% reduction (mild reduction)

25 patients showed 11-20% reduction (moderate reduction)

4 patients showed 21-30% reduction (moderate to severe reduction)

12 patients showed >=30% reduction (severe reduction)

The NPV values showed a Pearson Correlation of 0.091 concerning age, which indicated only a slight correlation but a negligible relationship. NPV values showed a P-value of 0.225 concerning age, which suggested a statistically non-significant relation (Table 4). The OPV values showed a Pearson Correlation of -.153 & a P-value of 0.041 concerning age, which denoted a statistically significant correlation. HPV values showed a significant Pearson Correlation of 0.391 concerning OPV at 0.05. TV values showed values of 0.634, 0.877 & 0.634 concerning NPV, OPV & HPV values, respectively, which denoted a moderate to high correlation with a marked relationship respectively & significant at 0.01 levels.

## Discussion:

The anatomy of the pharyngeal space is critical for effective swallowing and respiration, with airway volume influenced by age, gender and anatomical factors such as the tongue's size and the palate's shape. Cone-beam computed tomography (CBCT) has become a valuable tool for assessing airway volume, offering a three-dimensional view that surpasses the limitations of two-dimensional lateral cephalograms [18]. In our study, we utilized CBCT scans and ITK-SNAP software to analyze volumes of the nasopharynx, oropharynx and hypopharynx, finding that nasopharyngeal volume (NPV) averaged 7.29 cm^3^, with males generally showing higher NPV and total airway volume (TV) values. We observed a reduction in airway volume in 81 patients, categorized by severity, although obstructive sleep apnea (OSA) status was not explicitly assessed. Interestingly, 39 patients exhibited increased airway volumes, often associated with anterior tongue position and a high-arched palate.

Gender-based analyses indicated that males had significantly larger airway volumes, which aligns with prior studies that link higher airway values in males to sleep-disordered breathing risks. We also noted a statistically significant correlation between OPV values with age & HPV values, which corroborated previous research. Our findings support the role of CBCT & segmentation software in offering precise measurements of airway structures that could be vital for early identification of risks related to reduce airway volume. This subsequently could aid in developing tailored treatment strategies for managing conditions like OSA, providing a radiological alternative for effectively assessing airway patency and pharyngeal space dimensions in the absence of polysomnographic/sleep studies [19, 20]. This study underscores the importance of CBCT in understanding and potentially addressing age- and gender-related airway changes in clinical practice. Although not originally part of the aims of our research, we hope that the classification of severity based on the percentage reduction in airway volume given by us aids significantly in OSA research & treatment planning in the coming days.

## Conclusion:

A retrospective study of 180 patients found that males had significantly higher nasopharyngeal volumes (NPV) than females, with a strong correlation between age and oropharyngeal volume (OPV). Age also showed a non-significant correlation with NPV but was significantly correlated with OPV. Total airway volume showed moderate correlations with NPV, OPV and HPV. Future research on sleep-disordered breathing and OSA risk assessment is needed.

## Figures and Tables

**Table 1:** Mean & standard deviation of numerical variables in our study

	**N**	**Minimum**	**Maximum**	**Mean**	**Standard Deviation**
Age	180	20	72	42.61	13.66
NPV	180	3	13.3	7.297778	2.1568898
OPV	180	2	19	8.422778	3.3820398
HPV	180	1.4	11.4	5.267778	2.0360619
TV	180	9.4	40.1	20.98167	5.5794863

**Table 2:** Standard deviation & p-values of inter-group comparison of airway analysis based on gender

	**Gender**	**N**	**Mean**	**Std. Deviation**	**Std. Error Mean**	**T value**	**p-value of the t-test**
NPV	M	90	7.842222	2.3278469	0.2453766	3.491	.002**
	F	90	6.753333	1.8270871	0.1925919		
OPV	M	90	8.705556	3.6418405	0.3838837	1.123	.094#
	F	90	8.14	3.0951865	0.3262613		
HPV	M	90	5.447778	2.1568927	0.2273565	1.187	.282#
	F	90	5.087778	1.9026196	0.2005537		
TV	M	90	22.02889	6.2393144	0.6576815	2.557	.014*
	F	90	19.93444	4.6329344	0.4883542		

**Table 3:** The inter-group comparison of analysis based on gender showed a statistically non-significant difference seen for the frequencies between the groups (p>0.05)

		**Gender**				
		**F**	**M**	**Total**	**Chi-Square value**	**P value of Chi-Square test**
Airway	INC	14	25	39		
	N	31	29	60	4.169	.124#
	R	45	36	81		
	Total	90	90	180		

**Table 4:** Bivariate correlations

		**Age**	**NPV**	**OPV**	**HPV**	**TV**
NPV	Pearson Correlation	0.091				
	P value	0.225				
	N	180				
OPV	Pearson Correlation	-.153*				
	P value	0.041	0			
	N	180	180			
HPV	Pearson Correlation	-0.01	0.11	.391**		
	P value	0.889	0.13	0		
	N	180	180	180		
TV	Pearson Correlation	-0.07	.634**	.877**	.634**	
	P value	0.384	0	0	0	
	N	180	180	180	180	
